# The global compendium of *Aedes aegypti* and *Ae. albopictus* occurrence

**DOI:** 10.1038/sdata.2015.35

**Published:** 2015-07-07

**Authors:** Moritz U. G. Kraemer, Marianne E. Sinka, Kirsten A. Duda, Adrian Mylne, Freya M. Shearer, Oliver J. Brady, Jane P. Messina, Christopher M. Barker, Chester G. Moore, Roberta G. Carvalho, Giovanini E. Coelho, Wim Van Bortel, Guy Hendrickx, Francis Schaffner, G. R. William Wint, Iqbal R. F. Elyazar, Hwa-Jen Teng, Simon I. Hay

**Affiliations:** 1 Spatial Ecology and Epidemiology Group, Department of Zoology, University of Oxford, South Parks Road, Oxford OX1 3PS, UK; 2 Wellcome Trust Centre for Human Genetics,University of Oxford, Oxford, UK; 3 Institute for Health Metrics and Evaluation, University of Washington, Seattle, USA; 4 Department of Pathology, Microbiology, and Immunology, School of Veterinary Medicine, University of California, Davis, CA, USA; 5 Center for Vectorborne Diseases, University of California, Davis, CA, USA; 6 Fogarty International Center, National Institutes of Health, Bethesda, Maryland 20892, USA; 7 Department of Microbiology, Immunology and Pathology, Colorado State University, Fort Collins, CO, USA; 8 National Dengue Control Program, Ministry of Health, Brasilia, DF, Brazil; 9 European Centre for Disease Prevention and Control, Stockholm, Sweden; 10 Avia-GIS, Zoersel, Belgium; 11 Environmental Research Group Oxford Ltd, Department of Zoology, South Parks Road, Oxford OX1 3PS, UK; 12 Eijkman-Oxford Clinical Research Unit, Jakarta, Indonesia; 13 Center for Research, Diagnostics and Vaccine Development, Centers for Disease Control, Taipei, Taiwan (ROC)

**Keywords:** Risk factors, Dengue virus, Ecological epidemiology, Entomology

## Abstract

*Aedes aegypti* and *Ae. albopictus* are the main vectors transmitting dengue and chikungunya viruses. Despite being pathogens of global public health importance, knowledge of their vectors’ global distribution remains patchy and sparse. A global geographic database of known occurrences of *Ae. aegypti* and *Ae. albopictus* between 1960 and 2014 was compiled. Herein we present the database, which comprises occurrence data linked to point or polygon locations, derived from peer-reviewed literature and unpublished studies including national entomological surveys and expert networks. We describe all data collection processes, as well as geo-positioning methods, database management and quality-control procedures. This is the first comprehensive global database of *Ae. aegypti* and *Ae. albopictus* occurrence, consisting of 19,930 and 22,137 geo-positioned occurrence records respectively. Both datasets can be used for a variety of mapping and spatial analyses of the vectors and, by inference, the diseases they transmit.

## Background & Summary

*Aedes aegypti* [=*Stegomyia aegypti* ^1^] and *Ae. albopictus* [=*Stegomyia albopicta* ^1^] are disease vectors for many important viral human diseases such as dengue, chikungunya and yellow fever^2–4^. Dengue is the most prevalent human arboviral infection causing approximately 100 million apparent annual infections with almost half of the world’s population at risk^5^. Dengue transmission now occurs in over 120 countries^6^, mostly in the tropics and sub-tropics. Chikungunya, another arthropod-borne virus, has caused over 2.5 million infections over the past decade and has more recently been spreading in the Americas and emerging in Europe, posing new challenges to health systems as it spreads into new areas, infecting naïve populations and consequently causing large outbreaks^7–10^. The disease burden of yellow fever was significantly reduced due to large-scale vaccination programs in the twentieth century but current estimates of 51,000–380,000 severe cases in Africa per year point to the continuing difficulty in fully controlling this virus^11^. As a result, there is growing interest in describing the global geographic distribution of both vector species to better understand the risk of transmission of these viruses.

*Aedes aegypti* is a predominantly urban vector, utilising the abundance of artificial containers as larval sites and feeding almost exclusively on humans^12^. *Aedes albopictus* can more often be found in peri-urban and rural environments, feeding readily on a variety of mammalian (including humans) and avian species^13^.

*Aedes* mosquito surveys are performed to better understand ecological and epidemiological aspects of the vectors as well as to assist disease surveillance and control^14–16^. Surveillance of *Aedes* can involve; (i) systematic household surveys that involve searching water-filled containers for larvae and pupae, (ii) the use of backpack aspirators and suction traps baited with a chemical lure and/or CO_2_ for the collection of adult mosquitoes or, (iii) using ovitraps placed strategically around a neighbourhood to collect mosquito eggs that can then be reared back in the laboratory for morphological identification or directly processed for molecular identification^17–19^.

The database described here contains information on the known global occurrences of the adults, pupae, larvae or eggs of *Ae. aegypti* and *Ae. albopictus* globally from 1960 and 2014.

**Figure 1:**
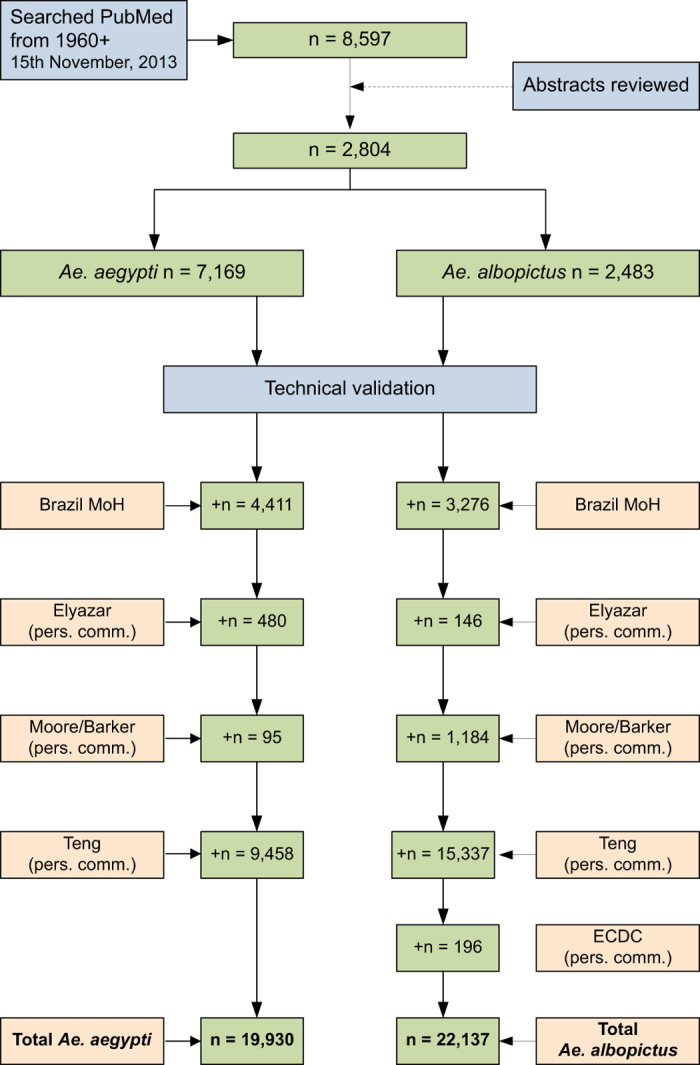
The numbers of occurrences for *Ae. aegypti and Ae. albopictus* by source.

By including data from a variety of sources we were able to create the largest currently available standardised up-to-date global dataset for both *Ae. aegypti* and *Ae. albopictus* (Fig. 1), containing 42,067 geo-positioned occurrences.

## Methods

### Data collection

PubMed (http://www.ncbi.nlm.nih.gov/nlmcatalog/journals) was searched using the term ‘*Aedes*’ OR ‘*aegypti*’ OR ‘*albopictus*’ for the years 1960 to 2013. The Medical Subject Headings (MeSH) term technology used in the PubMed citation archive ensured all pseudonyms were automatically included (http://www.nlm.nih.gov/mesh) in the searches. The same process was repeated for ISI Web of Science (http://wok.mimas.ac.uk) and ProMED (http://www.promedmail.org). The searches were last updated on 15th November 2013. No language restrictions were placed on these searches; however, only those citations with a full title and abstract were retrieved. This resulted in a collection of 8,597 references, of which 2,804 unique articles were identified from their abstracts as potentially containing useable location data. In-house language skills allowed processing of all English, French, Portuguese and Spanish articles. Confirmed *Aedes* occurrences within these articles were entered into the database. Occurrences were classified as confirmed when the article clearly stated the presence of the vector at a specific time in a specific location. This includes transient populations, i.e., found in ports or only during the summer months. Only for Europe were we able to include information of transient versus established populations using expert opinions. Laboratory studies were included if the mosquito/larvae were collected from the wild specifically for the purpose of the study. Occurrences were recorded separately for both species. More specific information about ‘sub-species’ or ‘genetic characteristics’ for example, were recorded where available but not included in the final database. This information can be obtained from the authors upon request. Data Citation 1 lists full references for each published record in the database.

In addition to the data directly sourced from published literature, primary and unpublished occurrence data from national entomological surveys were obtained through contact with administrators of these surveys when possible (Fig. 1). This includes primary data for *Ae. albopictus* provided from an earlier published article by Carvalho *et al.* ^20^ Collections have been part of the Levantamento Rapido de Indice para *Aedes aegypti* (LIRAa) in Brazil and are described in full elsewhere^20,21^. Similarly, *Ae. aegypti* primary data with geographic locations were also provided by an entomological survey directly from the Ministry of Health of Brazil for 2013. All occurrences for Brazil were classified as polygons (see geo-positioning methods) as they represent surveys conducted in Brazilian municipalities with their respective centroids being used as geographic information in the database. Primary and unpublished occurrence points for both species were provided by Elyazar (2014) for Indonesia. Moore and Barker (2014) provided summary data from the United States, collected between 1985–2014 from published and unpublished sources, including geographic location at the USA county administrative level which were included as polygons in the database. In addition, Teng (2014) added occurrence records for both species based on national entomological surveys performed in Taiwan between 2004–2013. European *Ae. albopictus* records were derived from the European Centre for Disease Prevention and Control (ECDC) funded TIGERMAPS and VBORNET datasets provided by Schaffner and Hendrickx (2013). Data were based on Nomenclature of territorial units for statistics 3 (NUTS3) level for Europe from 1979–2013. NUTS3 centroids were calculated in ArcGIS, occurrences classified as polygons and added to the database.

### Geo-positioning of data from published sources

All available location information was extracted for each occurrence from the relevant primary research article. The site name was used together with all contextual information provided about the site position to determine its latitudinal and longitudinal coordinates using Google Maps (https://www.maps.google.co.uk), Google Earth (http://www.google.co.uk/intl/en_uk/earth), or other online geo-positional databases including Geonames (http://www.geonames.org), Fallingrain (http://www.fallingrain.com/world/index.html) or as a last resort, using simple Google searches. Place names are often duplicated within a country, so contextual information was used to ensure the right site was selected. When the site name was not found, information from the text, was also used to scan sites in the approximate area to check for alternate spelling of the site name. If the study site could be geo-positioned to a specific latitude and longitude within a 5 km×5 km pixel, it was termed a ‘point location’. For each occurrence that could not be assigned a single 5 km×5 km pixel, e.g., a large city, the occurrence was entered as a polygon data type. Polygon occurrences were subsequently classified based on the polygon size they correspond to as either between 5–10 km^2^, 10–25 km^2^, 25–100 km^2^ or >100 km^2^. All locations were then linked to administrative units as recognised by the FAO Global Administrative Unit Layer (GAUL) system^22^. This initial database then underwent spatial and temporal standardisation and finally technical validation.

### Occurrence database management: spatial and temporal standardisation

As the database was compiled from many different sources and several institutions, it was first necessary to standardise the data entries such that identical locations which may have been geo-positioned slightly differently were given the same unique identifier. Point records were given the same unique identifier if they lay within the same 5 km×5 km pixel within a global grid. Finally, any record associated with a polygon measuring larger than 111 km×111 km at the equator (1 degree) was removed from the database (*n*=475, *n*=54 for *Ae. aegypti* and *Ae. albopictus* respectively).

Similarly, it was necessary to temporally standardise the database to avoid duplicates. We chose to define a single occurrence at a given unique location (as identified above) within one calendar year. This was particularly important for oversampled regions that undergo multiple yearly surveys such as Taiwan and involved a procedure which: (i) disaggregated any records which were in the same location but spanning multiple years into individual records for each respective year and then (ii) aggregated all records with the same unique location identifier and occurring within the same year to form a single occurrence record. This led to 1,112 and 370 records being removed for *Ae. aegypti* and *Ae. albopictus,* respectively.

## Data Records

This database is publicly available online as a comma-delimited file for both species independently for ease of use and the ability to import it into a variety of software programs (Data Citation 1). Each of the rows represents a single occurrence record (one or more *Aedes* cases in the same unique location within a single calendar year). The fields contained in the database are as follows:

1. **VECTOR:** Identifying the species; *Ae. aegypti* or *Ae. albopictus*
2. **OCCURRENCE_ID:** Unique identifier for each occurrence in the database after temporal and spatial standardisation.
3. **SOURCE_TYPE:** Published literature or unpublished sources with reference ID that corresponds to the full list of references in Data Citation 1.
4. **LOCATION_TYPE:** Whether the record represents a point or a polygon location.
5. **POLYGON_ADMIN:** Admin level or polygon size which the record represents when the location type is a polygon. −999 when the location type is a point (5 km×5 km).
6. **X:** The longitudinal coordinate of the point or polygon centroid (WGS1984 Datum).
7. **Y:** The latitudinal coordinate of the point or polygon centroid (WGS1984 Datum).
8. **YEAR:** The year of the occurrence.
9. **COUNTRY:** The name of the country within which the occurrence lies.
10. **COUNTRY_ID:** ISO alpha-3 country codes.
11. **GAUL_AD0:** The country-level global administrative unit layer (GAUL) code (see http://www.fao.org/geonetwork) which identifies the Admin-0 polygon within which any smaller polygons and points lie.
12. **STATUS:** Established versus transient populations.

## Technical Validation

The following procedures were carried out on the final database to ensure the accuracy and validity of the occurrence records.

1. A raster distinguishing land from water^22^ was created at a 5 km×5 km resolution and was used to ensure all occurrences were positioned on a valid land pixel (*n*=95 and *n*=64 records were removed for *Ae. aegypti* and *Ae. albopictus* respectively).
2. We cross-validated all of the unique occurrence locations against temperature-based *Aedes* population persistence metrics developed by Brady *et al.* ^23^ In brief, this classification was determined by modelling the effect of temperature on adult *Ae. aegypti* and *Ae. albopictus* survival and length of first gonotrophic cycle, the interaction of which determines whether the population can persist. Population persistence was then predicted on a global scale using interpolated meteorological data^24^. Occurrences that fell outside this range were re-checked to ensure the quality of the occurrence records.

**Figure 2:**
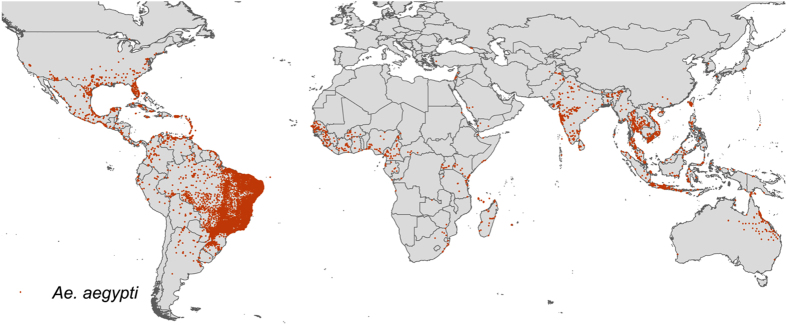
Map of occurrence points for *Ae. aegypti*.

**Figure 3:**
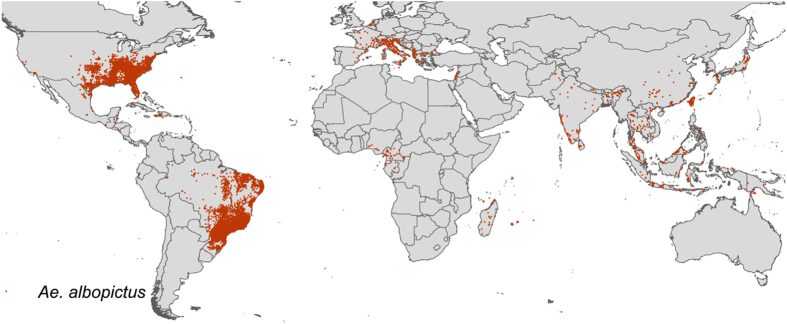
Map of occurrence points for *Ae. albopictus.*

**Figure 4:**
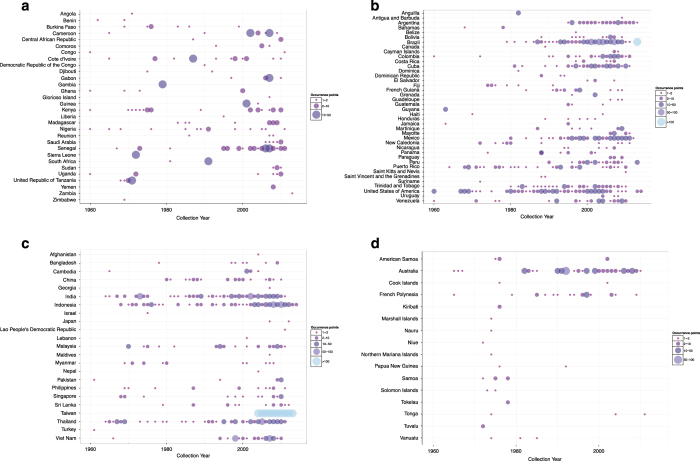
The numbers of occurrences for *Ae. aegypti* with locations per year separated by region, with panels (**a**–**d**) representing regions. Colours indicate the number of mosquitoes recorded in each year with the size of the circles scaled respectively.

**Figure 5:**
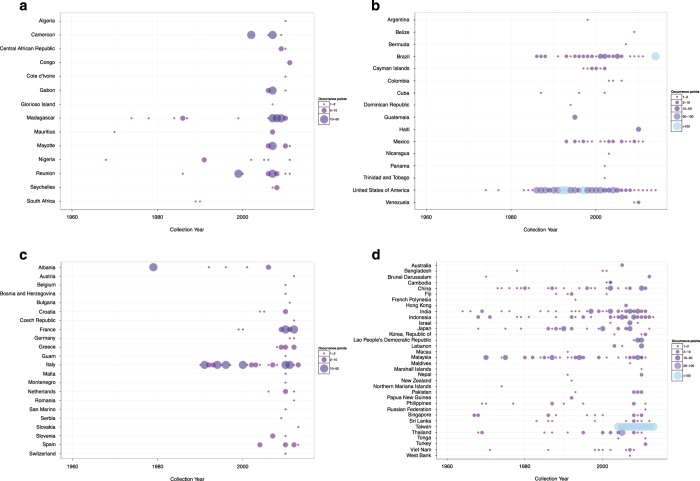
The numbers of occurrences for *Ae. albopictus* with locations per year separated by region, with panels (**a**–**d**) representing regions. Colours indicate the number of mosquitoes recorded in each year with the size of the circles scaled respectively.

The result is a database consisting of 19,930 and 22,137 geo-positioned occurrences in total worldwide for *Ae. aegypti* and *Ae. albopictus* respectively, broken down by region, location type and source type in Fig. 1. In Figs 2 and 3 the global geographic distribution of both species is displayed. Figs 4 and 5 show occurrence records by year and region for both *Ae. aegypti* and *Ae. albopictus* respsectively. The increase in occurrence records since 2013 is largely attributable to the LIRAa data from Brazil.

## Usage Notes

The dataset described here can be used to investigate the spatial and temporal patterns of *Aedes* distribution at multiple scales and resolutions. As *Ae. aegypti* and *Ae. albopictus* are invasive species, spreading to new areas via shipping routes and human movement^25–27^, this dataset could improve predictions of locations at high-risk for importation^25^. This dataset can also be used to contribute to modelling areas at risk for dengue^28^ and chikungunya^9^ especially in areas in Europe^16,28^ and the USA^29,30^. We aimed at building a comprehensive set of data based on occurrences ever recorded globally including their respective dates to allow researchers as well as policy makers to filter the dataset based on their respective research questions.

This dataset was first used in an ecological niche modelling framework along with a set of environmental covariates to map the global distribution of each species^32^. A generic code to produce the global risk maps is openly available as an R software package ‘seegSDM’ from GitHub (https://github.com/SEEG-Oxford/seegSDM). Such maps can help to guide vector surveillance efforts in countries where the distribution of both species is not well-known, but which are at high risk for importation of related viruses.

Regional biases in density of occurrence records are apparent and may be due to differences in the amount of regular surveillance, differences in the number of published studies and availability of routinely collected data. Use on a global scale, however, would need to take into account geographical sampling bias as done in Kraemer *et al.* using similarly biased background points in a presence-only niche modelling approach^31,32^. The method for accounting for sampling bias, however, might vary depending on the research question asked and methodology applied in subsequent analyses.

## Additional Information

**How to cite this article:** Kraemer, M. U. G. *et al.* The global compendium of *Aedes aegypti* and *Ae. albopictus* occurrence. *Sci. Data* 2:150035 doi: 10.1038/sdata.2015.35 (2015).

## Supplementary Material
